# Evaluating a Research Training Program in Environmental Health and Noncommunicable Diseases in Georgia

**DOI:** 10.3390/ijerph22091433

**Published:** 2025-09-14

**Authors:** Carla J. Berg, Lela Sturua, Amiran Gamkrelidze, Tina Beruchashvili, Tinatin Manjavidze, Givi Javashvili, Nino Kiladze, Levan Baramidze, W. Michael Caudle

**Affiliations:** 1 Department of Prevention and Community Health, Milken Institute School of Public Health, George Washington University, Washington, DC 20052, USA; 2 GW Cancer Center, George Washington University, Washington, DC 20052, USA; 3 Noncommunicable Diseases Department, National Center for Disease Control and Public Health, Tbilisi 0179, Georgia; lela.sturua@ncdc.ge; 4 Department of Public Health and Healthcare Management, School of Health Sciences, University of Georgia, Tbilisi 0175, Georgia; am.gamkrelidze@ug.edu.ge (A.G.); t.beruchashvili@ug.edu.ge (T.B.); t.manjavidze@ug.edu.ge (T.M.); 5 International School of Public Health, Tbilisi State Medical University, Tbilisi 0177, Georgia; gjavashvili@tsmu.edu (G.J.); l.baramidze@tsmu.edu (L.B.); 6 Department of Hygiene, Medical Ecology and Health Promotion, Tbilisi State Medical University, Tbilisi 0177, Georgia; nkiladze@tsmu.edu; 7 Gangarosa Department of Environmental Health, Rollins School of Public Health, Emory University, Atlanta, GA 30322, USA; william.m.caudle@emory.edu

**Keywords:** global health training, global health, mentorship, environmental health, noncommunicable diseases, low- and middle-income countries

## Abstract

The Clean Air Research and Education (CARE) program, launched in 2020, aims to enhance environmental health (EH) and noncommunicable disease (NCD) research capacity in the Republic of Georgia. This paper evaluates the first 4.5 years of CARE, summarizing fellows’ activities and achievements to date and fellow and faculty reactions to CARE. In February 2025, CARE leadership anonymously surveyed fellows (100% response rate: *n* = 23/23; 4 Master’s of Public Health [MPH], 19 PhD) and faculty (66.7%: *n* = 10/15; 6 Georgia-based, 4 US-based). Thesis/dissertation topics included tobacco (43.5%), air pollution and respiratory outcomes (each 21.7%), lead exposure and cancer-related and cardiovascular outcomes (each 13.0%), and others. Fellows leveraged CARE’s financial support for research execution (78.3%), scientific conferences (34.8%), specific training (21.7%, *n* = 5/23), and/or publication fees (26.1%). Fellows indicated that the most valuable program aspects were opportunities for (1) building/expanding professional networks; (2) exposure to experts and training; and (3) instrumental support to pursue their PhD and conduct research. Fellows and faculty prioritized sustaining the following: structured mentor–mentee relationships; involvement of US-based mentors; support identifying research funding and preparing publications; and training in methods/data analysis. This study provides a model for evaluating other research training programs and highlights the important role such programs may play in developing the capacity to conduct relevant public health research in low- and middle-income countries.

## 1. Introduction

The mounting burden of noncommunicable diseases (NCDs) in low- and middle-income countries (LMICs) was recognized over two decades ago by the World Health Organization (WHO) [1]. In 2021, NCDs were responsible for ≥43 million people (~75% of non-pandemic-related deaths globally), with 18 million NCD-related premature deaths [2]. Of all NCD deaths, 73% are in LMICs, including 82% of premature deaths [2,3,4]. Cardiovascular diseases account for most NCD deaths, followed by cancers, chronic respiratory diseases, and diabetes; these diseases account for 80% of all premature NCD deaths [2,3,4]. Key risk factors for NCDs include air pollution, tobacco use, physical inactivity, unhealthy diets, and others [2,3,4]. Notably, almost all of the global population (99%) breathes air that exceeds WHO limits and contains high levels of pollutants, with LMICs having the highest exposures [5,6].

Training programs in LMICs are critical for building global research capacity and addressing particular health needs in LMICs. One of the most prominent leaders in this area is the US National Institutes of Health, particularly Fogarty International Center (FIC). For >50 years, FIC has built research capacity, particularly in LMICs, providing training for >6000 health scientists from >100 countries [7]. Programs funded by FIC and other centers and institutes within the US National Institutes of Health (NIH, including the National Institute of Environmental Health Sciences [NIEHS]) aim to address international health priorities and enhance institutional capacity for research in environmental health (EH), NCDs, and a range of other health-related topics (e.g., infectious diseases, injury, health systems implementation, medical education, and research ethics) [7].

One prominent FIC funding mechanism for such programs is the D43 grant, specifically designed to establish partnerships between foreign and US-based institutions to enhance research capacity in LMICs and ultimately strengthen global health research [8,9]. Such global health training programs involve formal graduate education, such as Master’s, doctoral, and postdoctoral degree programs related to the training areas needed and health concerns within fellows’ home countries [10,11]. Prior evaluations of such programs have shown that alumni are highly successful and yield desired outcomes. One evaluation of 257 FIC alumni indicated that most remained engaged in LMICs (63%), worked in academic/research careers (70%), and/or received new grants as principal investigator (PI), co-/multi-PI, or site PI (56%), with 438 new grants and 5318 publications represented among them [12]. These training programs highlight the value of LMIC research experience in nurturing the global health research workforce.

This paper focuses on a NIEHS/FIC-funded D43 in the Republic of Georgia. Launched in 2020, the Clean Air Research and Education (CARE) program is a collaboration between Emory University, the Georgia National Centers for Disease Control and Public Health (NCDC), Tbilisi State Medical University, University of Georgia in Tbilisi, and George Washington University. CARE has the long-term goal of enhancing capacity in Georgia to conduct research related to EH and NCDs, and ultimately to inform related policy and practice [13]. In Georgia, ~94% of all deaths are due to NCDs [14], and Georgia’s mortality index attributed to ambient and indoor air pollution is >200, the third highest in the world [15]. Accordingly, Georgia’s National Health Action Plan, which is conceptually and strategically linked with the United Nations’ 2030 Sustainable Development Goals and Health 2020, highlights that NCDs and air pollution are among the most prominent public health priorities.

Despite the importance of addressing EH and NCDs in Georgia, there is limited in-country capacity to conduct research regarding the impact of such environmental hazards on NCD-related health outcomes. Moreover, only a couple of research training programs have existed in Georgia [16,17] or this region in general [18,19,20], as a particularly larger proportion have been placed in Africa or Asia [21]. Thus, the CARE program aims to address these gaps via EH- and NCD-related training and mentored research opportunities for Master’s of Public Health (MPH) and PhD trainees in Georgia.

Furthermore, few research training programs have a specific focus on knowledge translation to inform policy and practice. CARE recognized the importance of ensuring research dissemination and knowledge translation to impact policy and practice [22], and the need to prepare fellows to serve as national public health leaders in their countries [7], especially given the small population of Georgians and of skilled public health researchers in Georgia, positioning fellows to advance to important public health leadership roles. Thus, enhancing dissemination and knowledge translation skills among fellows is an explicit goal of CARE. Moreover, the critical time period of this training program—which spanned from just before (2020) to after (2025) the COVID-19 pandemic—offered opportunities to enhance certain training opportunities in response to related public health needs.

This paper provides data regarding the evaluation of the first 4.5 years of CARE. One prior evaluation of CARE (conducted in 2022 after the first 1.5 years of the program) involved only the initial 12 fellows (4 MPH and 8 PhD students) and assessed initial challenges faced during the launch of the program, particularly within the context of the COVID-19 pandemic [23]. This evaluation emphasized challenges, including the following: (1) disruptions to training and networking opportunities as program leadership reconsidered channels for training and meetings; (2) difficulty meeting program demands as fellows and faculty were also committed to careers in public health; and (3) an evolution of public health priorities [23]. Based on this evaluation [23], CARE leadership implemented increased communication among program leadership, faculty/mentors, and fellows, and also chose to focus on advanced training at the PhD level, with less emphasis at the MPH level, to respond to the increased need for such skills at this more advanced level within Georgia [24,25,26,27].

The current evaluation (1) involves a larger number of fellows; (2) includes assessment of a more mature training program, including more training activities (informed by the prior evaluation and to meet timely public health priorities post-pandemic) and longer-term mentored research experiences, over a longer period of time; and (3) uses a mixed-methods approach, integrating qualitative and quantitative methods. The current evaluation aimed to describe short- to intermediate-term outcomes of the training program, specifically by (1) assessing the utilization of CARE program opportunities (e.g., training, research support); (2) characterizing fellows’ thesis/dissertation research (i.e., topics, study designs, dissemination outcomes); and (3) evaluating fellow and faculty reactions to CARE (i.e., importance of program components, impact). Ultimately, this paper aims to contribute empirical data related to the implementation and early outcomes of research training in a post-Soviet LMIC, which is relevant for funders, policymakers, and universities in similar regions and globally.

## 2. Materials and Methods

### 2.1. Training Program Description

CARE was funded in September 2019, accepted its first cohort of MPH trainees (*n* = 2) in September 2020, and subsequently enlisted 2 additional MPH students and 19 PhD students (i.e., 4 MPH students total—all at Tbilisi State Medical University; 19 PhD students total—5 at Tbilisi State Medical University and 14 at University of Georgia in Tbilisi). Notably, this program has been progressive in its recruitment of trainees, as other training programs have generally had fewer trainees within the first 5 years of the training program’s existence. For example, one D43 training program in Kenya proposed 2 PhD and 4 Master’s students in a 5-year period [28], another in Tanzania planned to train 5 PhD and 5 Master’s students (10 total) in 4 years [29], a separate D43 training program in Georgia trained 31 fellows over 16 years (~10 per 5-year period) [30], and one in South Africa trained 44 fellows in 10 years (i.e., ~22 per 5-year period) [31]. Thus, despite the seemingly small sample, it represents a relatively large training program.

CARE enhances research capacity via meetings/workshops, formal didactic trainings, mentorship, mentored research, and other instrumental research support [23]. Table 1 provides an overview of activities since the program’s launch. Supplementary Figure S1 provides an overview of the conceptual framework for CARE’s training in EH and NCD research; Supplementary Figure S2 depicts CARE’s evaluation framework. This evaluation was conducted in February, 2025, and was deemed exempt by the George Washington University Institutional Review Board (as these data were collected as an evaluation of educational programming).

#### 2.1.1. Semi-Annual Care Meetings

Semi-annual meetings, held in each spring and fall, entail the following: orientation for new fellows and faculty, comprehensive training in responsible conduct of research, fellows’ presentations of their proposed and ongoing research, keynote lectures (e.g., public health communication), and workshops on special topics (e.g., global health diplomacy, social determinants of health, state of global environmental health sciences) and related to professional development (e.g., mentorship, communication, presenting and publishing research findings). (Each meeting also includes workshops/seminars on responsible conduct of research; see below.)

#### 2.1.2. Formal Training

In the initial program implementation phases, CARE program leadership (comprising researchers at Emory, George Washington University, NCDC, Tbilisi State Medical University, and University of Georgia) worked with each Tbilisi-based university to assess EH and NCD research training needs. Careful review of curricula at both institutions found that NCDs were covered in various courses, and course content was augmented to ensure robust coverage of key NCD-related topics. Other training needs included (1) key EH topics, including NCD implications; (2) research methods and data analysis; and (3) research dissemination and knowledge translation to impact policy and practice. These core needs were addressed through (1) an annually provided course in EH; (2) seminars covering qualitative and quantitative research methods (to supplement existing curricula) and a course on advanced data analysis and big data; and (3) two courses related to policy and practice—(a) global health diplomacy and (b) public health emergency preparedness. Additionally, training in responsible conduct of research was provided via didactics (e.g., history/principles of research ethics, protecting vulnerable groups, informed consent, conflicts of interest, research misconduct, authorship, effective mentor–mentee relationships, data management/security, ethics in scientific writing) and group discussion during CARE meetings and other opportunities (e.g., Fellow Club meetings, ongoing mentorship—described below). See Table 1 for the timeline of courses/trainings.

#### 2.1.3. Mentorship and Mentored Research Activities

Mentored research activities are designed to coincide with faculty EH- and NCD-related research, and to support fellow-led studies. Mentorship is provided via pairs of mentors from each country. Mentors were identified by CARE program leadership to represent content area and methodical expertise to match the fellows’ interests, and then contacted to request their engagement (which was favorably received by the faculty invited).

Fellows and their mentorship teams are asked to arrange monthly to bimonthly meetings. Although data were not collected on actual dates/times met, the semi-annual CARE meetings entail such mentor–mentee sessions; thus, fellows and their mentors meet at least twice annually (at the semi-annual CARE meetings), and as needed.

Because of the importance of effective mentorship, several activities aim to enhance related experiences and skills among fellows and faculty, including sessions focused on successful mentor–mentee relationships, communication skills, etc., during semi-annual CARE meetings, monthly Fellow Club meetings, and quarterly meetings among faculty mentors [32,33,34,35,36,37,38].

#### 2.1.4. Monthly CARE Fellow Meetings

Fellows participate in monthly Fellow Club meetings. These meetings have covered special topics (e.g., imposter syndrome, mentorship, communication), fellow updates on their research projects, and research presentations by Georgia- and US-based faculty.

#### 2.1.5. Other Instrumental Support to Conduct Research

Funds are available to support fellows’ thesis/dissertation research, publication fees, participation in scientific conferences, or specific training outside of their home institution, etc. To access these funds (ranging up to USD 5000 unless otherwise justified due to unusual/unexpected circumstances), fellows are required to provide to program leadership a proposal outlining the anticipated expenses, the rationale, etc., and to provide thorough documentation of all funds utilized.

### 2.2. Measures

In February 2025, program leadership administered an online survey to fellows and faculty mentors to evaluate the initial 4.5 years of the program, including its overall activities, training, and resources. The survey was adapted from previously published training program evaluations (including others’ [39,40] and our own [23]; see Supplementary File S1) and pilot-tested by 4 MPH-level research assistants prior to launching data collection. All fellows and faculty were contacted (i.e., 23 fellows, 15 faculty); note that this represents all fellows admitted into the program, as no trainees left the program. Participants were given four weeks to respond to the survey and were provided with two weekly prompts via email and text message during the first three weeks, and via email and phone calls in the final week.

#### 2.2.1. Participant Characteristics

The survey assessed participants’ gender, years in the program, institutional affiliation, and MPH versus PhD track among fellows.

#### 2.2.2. Thesis/Dissertation Characteristics

The survey assessed characteristics of fellows’ thesis/dissertation research, including data sources (i.e., primary/secondary data; surveys, focus groups, etc.); study design; topics; and populations (see Supplementary File S1). Fellows were also asked, “During CARE, indicate whether you have had email, telephone, or in-person communication with the following people or entities to discuss your CARE project: other scientists affiliated with CARE; other scientists not affiliated with CARE; government officials (e.g., Ministry of Health); community partners (e.g., local community health agencies); local non-governmental organizations or other advocacy organizations.”

#### 2.2.3. Training and Meeting Evaluations

For each class (EH, Global Health Diplomacy, Research Methods, Data Analysis, Responsible Conduct of Research), fellows were asked to rate their perceptions of the course across four dimensions (level of learning, stimulation, relevance to career goals, instructor knowledge and presentation; 1 = strongly disagree to 5 = strongly agree). Similar items assessed the CARE Meetings and Fellow Club (see Supplementary Table S1).

#### 2.2.4. CARE Resource Utilization, Importance, and Impact

Fellows were asked, “So far, what CARE resources have you used—funds to support: conducting research, attending conferences, specific trainings outside of your university, and publication fees.” Fellows were asked how helpful or important each of the CARE resources were (1 = not at all to 4 = a lot), and the extent to which they agreed with statements regarding CARE’s impact (1 = strongly disagree to 5 = strongly agree). Fellows and faculty were asked which CARE components were most crucial to sustain beyond the grant funding period (see Supplementary File S1).

#### 2.2.5. Qualitative Evaluation of CARE

Fellows and faculty were asked the following open-ended questions: (1) What have you learned about yourself from being a mentor or mentee in CARE? (2) What about CARE has been most valuable to you? (3) If a funder were to ask you why CARE or similar programs are important to your country, what would you say? (4) If a funder were to ask you why CARE or similar programs are important to global health, what would you say? and (5) What would you suggest or change about this overall CARE program?

### 2.3. Data Analysis

Descriptive analyses were conducted using SPSS v26 (IBM, Armonk, NY, USA) to characterize the fellow and faculty participants and responses to closed-ended items. Inductive thematic analysis was used to analyze responses to open-ended questions. Specifically, two members of program leadership systematically reviewed an initial subset of five responses from fellows and five responses from faculty to each open-ended question, and identified preliminary themes that emerged. They then compared the preliminary themes identified, and reconciled thematic codes before reviewing and coding the subsequent responses. They then selected representative quotes to summarize key themes.

## 3. Results

### 3.1. Participant Characteristics

Response rates were 100% (*n* = 23/23) for fellows and 66.7% (*n* = 10/15) for faculty. Table 2 summarizes participant characteristics.

### 3.2. Fellows’ Thesis/Dissertation Characteristics

As shown in Table 3, thesis/dissertation topics included tobacco-related topics (43.5%, *n* = 10/23); air pollution and respiratory outcomes (each 21.7%, *n* = 5/23); lead exposure and cancer-related and cardiovascular outcomes (each 13.0%, *n* = 3/23); and water/sanitation/hygiene, nutrition, reproductive and mental/cognitive health outcomes, and toxicology (each 8.7%, *n* = 2/23). Target populations included the general adult population (47.8%, *n* = 11/23), women (26.1%, *n* = 6/23), children (21.7%, *n* = 5/23), and men, clinical/patient populations, healthcare providers, and specific geographic populations (each 13.0%, *n* = 3/23).

Shown in Table 4, thesis/dissertation research involved primary data collection (47.8%, *n* = 11/23), secondary data analysis (26.1%, *n* = 6/23), or both (26.1%, *n* = 6/23). Primary data collection efforts included surveys (43.5%, *n* = 10/23) among children, patients, physicians, street food consumers, school-aged students, college students, and teachers; biological assessments (13.0%, *n* = 3/23), including allergy tests and pregnant women’s salivary cortisol assessments; and qualitative data collection using focus groups (13.0%, *n* = 3/23) and semi-structured interviews (4.3%, *n* = 1/23) among pregnant women, teachers, and students. Secondary data were from the National Environmental Agency (air pollutants/pollution levels); cancer registry; birth registry; state and regional lead exposure databases; NCDC all-cause mortality datasets; and medical charts. Study designs were commonly cross-sectional (69.6%, *n* = 16/23), longitudinal (26.1%, *n* = 6/23), and case control studies (17.4%, *n* = 4/23).

Many fellows established communication with other scientists within (47.8%, *n* = 11/23) and outside of CARE (34.8%, *n* = 8/23), government officials (30.4%, *n* = 7/23), and community partners (34.8%, *n* = 8/23; Table 4).

### 3.3. Fellows’ Use of CARE Resources

Shown in Table 5, 78.3% (*n* = 18/23) of fellows leveraged CARE’s financial support to conduct thesis/dissertation research, 34.8% (*n* = 8/23) to attend scientific conferences, 26.1% (*n* = 6/23) for publication fees, and 21.7% (*n* = 5/23) to obtain specific training outside of their home university.

### 3.4. Fellows’ Reactions to CARE Resources

Supplementary Table S1 shows fellows’ ratings of training and activities. All were rated highly, especially the EH course (M = 4.77, SD = 0.68), global health diplomacy course (M = 4.64, SD = 0.89), responsible conduct of research training (M = 4.57, SD = 0.69), CARE meetings (M = 4.51, SD = 0.93), and data analysis course (M = 4.50, SD = 1.04).

CARE resources deemed most helpful or important (Table 5) were funds for thesis/dissertation research, scientific conferences, and training outside of the home university (all M = 4.0), followed by mentorship from Georgia- and US-based mentors (M = 3.96, SD = 0.20), funds for publication fees (M = 3.89, SD = 0.33), and additional training in research/analytic methods (M = 3.87, SD = 0.34).

As shown in Table 6, among both fellows and faculty, program components most commonly endorsed as important to sustain were structured mentor–mentee relationships (95.7% among fellows and 80.0% among faculty); involvement of US-based mentors (95.7% and 70.0%); instruction/support for finding research funding (91.3% and 70.0%) and preparing publications, abstracts, presentations, etc. (91.3% and 70.0%); and enhanced training in research methods/data analysis (65.2% and 90.0%).

### 3.5. Overall Impact of CARE

Fellows’ responses were mostly positive in assessing CARE’s impact on enhancing research skills (M = 4.75, SD = 0.44), exposing fellows to learning important for their career (M = 4.71, SD = 0.55) or that they would not have otherwise had (M = 4.63, SD = 0.65), enhancing their interest in EH and NCDs (M = 4.63, SD = 0.65), and enhancing mentors’ investment in their careers (M = 4.63, SD = 0.65; Table 6).

Below, qualitative themes (from open-ended questions) are summarized and representative quotes are presented; Supplementary Table S2 shows additional representative quotes. Through participating in the program, fellows and faculty reportedly learned the importance of (1) being resourceful, adaptable, and resilient in conducting research and solving problems; (2) continuous learning and personal/professional development; and (3) collaborations and supporting one another. For example, one fellow wrote, “I realized that with the right guidance and support, I can overcome obstacles and improve my research skills. I have become more organized and better at solving problems, which helps me work through research challenges. I also learned to be more patient and to see difficulties as learning opportunities rather than failures.” Another fellow stated, “It has taught me the importance of resilience, adaptability, and continuous learning. I’ve discovered my ability to tackle complex problems, manage challenges, and work collaboratively with diverse teams. It has also helped me recognize areas for personal growth, particularly in leadership and time management, and has strengthened my commitment to pursuing impactful research.” One faculty member indicated, “It was a unique chance to explore new approaches to public health and gain new knowledge about important environmental health issues and challenges. The process of collaborating with colleagues was the most important benefit in this project.”

Fellows and faculty indicated the particular importance of certain resources and opportunities, including (1) the opportunity to build/expand their professional network of researchers and other stakeholders; (2) exposure to experts and up-to-date evidence and training; and (3) instrumental support necessary for many fellows to be able to pursue their PhD and conduct their own research. One fellow stated, “[The most valuable aspect of the program has been] exposure to cutting-edge research and the opportunity to collaborate with experts in global health and environmental studies….”. Another fellow stated, “Without the support of the CARE program, I wouldn’t have had opportunity to pursue a PhD.” A faculty member stated, “It has been a hands-on learning experience that the environmental health issues are truly global but may manifest in different ways given the international circumstances.”

Themes regarding CARE’s importance to Georgia related to particular challenges in Georgia given its sociopolitical history as a former Soviet Union country; its status as an LMIC; gaps in expertise and infrastructure to conduct public health research; and the need to address challenges and gaps to make meaningful contributions to advancing global health. One fellow stated, “Programs like this are crucial for strengthening Georgia’s capacity to conduct high-quality research. As a small country, Georgia benefits from partnerships that provide access to international expertise, resources, and cutting-edge methodologies, enabling local researchers to address both national and global health challenges effectively.” One faculty member indicated, “Investing in such initiatives is crucial for developing a strong public health workforce that can drive sustainable improvements in healthcare and population health in Georgia.”

Themes related to CARE’s importance for global health in general involved the need for global health research capacity in all countries, including LMICs that may face particular health challenges that are relevant to other locations. One fellow summarized eloquently, “This program and similar initiatives are essential to global health as they provide researchers from around the world with the tools, knowledge, and support needed to tackle complex health issues that transcend borders.”

Both fellows and faculty made statements indicating that the program met specific fellow needs. However, suggested improvements among fellows included the following: (1) post-program support for fellows (e.g., to support their research, facilitate ongoing collaborations with their US-based mentors and other researchers); (2) providing more exposure to a more diverse group of experts across disciplines (e.g., policy, economics); and (3) more time/opportunities for applied research, grant/scientific writing, etc. Faculty commonly commented on the need for more time allocated for fellows to focus on and complete their thesis/dissertation research.

### 3.6. Summary of Early Achievements Related to Program Outcomes

Supplementary Table S3 provides an overview of the short-, intermediate-, and long-term outcomes of the CARE program and related achievements to date. Short-term achievements include (1) increasing fellow knowledge/skills in EH, NCDs, methods/analysis, responsible conduct of research, and dissemination and translation to policy and practice via successful completion of all courses/trainings and demonstration in conducting research among all fellows; and (2) increasing mentored trainees, mentors, and mentor research projects, as well as facilitating degrees earned (i.e., 4 MPH students graduated [all within the expected timeframe], all PhD students are on track to meet their timelines).

Intermediate achievements include (1) increasing EH/NCD research as indicated by the increased number of peer-reviewed publications (including 2 of 4 completed MPH theses published and ~18 dissertation papers published/accepted from 9 PhD students to date) and abstracts (~24 to date); and (2) enhancing career development/promotion, as many fellows are serving in high-level positions and earning promotions in organizations under the Ministry of Health (e.g., National Centers for Disease Control and Public Health, National Environmental Agency), and are assuming professor/instructor positions in public health at Tbilisi-based universities.

Longer-term outcomes will require additional time to obtain data reflecting the realization of these goals. However, CARE has made strides in (1) enhancing infrastructure and capacity for high-quality research on EH and NCDs by enhancing public health curriculum in two premier public health universities (additional content/courses in NCDs, EH, global health diplomacy, emergency preparedness, methods/analysis), and by enhancing mentorship skills of faculty and fellows; and (2) fostering the development of a critical mass of EH/NCD researchers and multidisciplinary collaborations in Georgia through network-building activities such as CARE meetings and attendance at scientific/professional meetings, and by engaging fellows and faculty representing a wide range of institutions, backgrounds, disciplines, and professional experiences—including from the broad spectrum of public health (e.g., EH, global health, epidemiology, health behavior sciences, biostatistics), law, public administration, etc.

## 4. Discussion

Building research capacity among health professionals has long been recognized as crucial to advance global health priorities and address health disparities between developing and developed countries [41,42], with NCDs and EH [2,6] representing two key global health priorities with particular disparities. This manuscript presented results from an early evaluation of the CARE program, which launched in 2020 and aims to build EH and NCD research capacity in Georgia.

These early results indicate successful advances among 4 MPH fellows and 19 doctoral fellows, despite early disruptions to the program, largely due to the COVID-19 pandemic [43]. The pandemic dramatically altered global programs to train researchers in LMICs and how NCDs and EH were addressed within the context of public health and societal challenges [43]. An assessment of CARE 1.5 years after program launch helped program leadership refine the program, including the nature of communication (e.g., virtual/distance learning [19], virtual and hybrid meetings), and shift to focusing more on PhD- (vs. MPH-) level training [23]. This approach has been acknowledged by other global health research training programs, based on the need to ensure that the programs yield researchers with the skills and expertise to lead research and public health initiatives in their countries [24,25,26,27]. This is crucial, as one evaluation among FIC alumni indicated the importance of FIC fellowships in establishing the careers of LMIC doctoral scholars, most of whom remained engaged and productive in global health research [12], advancing the health of their home countries and serving as mentors to additional future leaders in public health research and practice [12]. Moreover, pandemic-related experiences also led CARE leadership to consider the framing of training related to dissemination and translation of research findings to inform policy and practice, resulting in two courses that were highly rated by fellows—global health diplomacy [44,45] and emergency preparedness [46,47] (led by the former director of the NCDC and involving several guest lecturers from key agencies within and outside of Georgia). Taken together, CARE represents a research training program with unique experiences, based on its timing and ability to respond to key opportunities in the context of pandemic-related challenges.

The activities and achievements of CARE fellows align with the goals of the program. Thesis/dissertation topics addressed diverse EH- and NCD-related topics (e.g., tobacco, air pollution, lead exposure), outcomes (e.g., cardiovascular, cancer, respiratory outcomes), and populations (e.g., general population, children, healthcare providers). Nearly 75% of fellows conducted primary data collection, and over half used secondary data sources (e.g., from NCDC or National Environmental Agency), emphasizing the crucial role of engaging these organizations and their key leaders in the program (e.g., as members of program leadership or mentors). Moreover, increasing overall research capacity requires a network of researchers with diverse methodological skills, and fellows used various study designs (e.g., cross-sectional, longitudinal) and diverse methods (e.g., surveys, qualitative assessments).

Fellows leveraged CARE’s resources to support the conduct of their research, dissemination of research results (via publication fees and attending scientific conferences), and ability to obtain specific training. Key themes regarding crucial aspects of the CARE program centered on access to expertise, mentorship, and networking opportunities, similar to findings from evaluations of other research training programs [9,48]. Particularly important to note about this program is its involvement of public health researchers, practitioners, and leaders across universities and key institutions under the Ministry of Health (e.g., NCDC, National Environmental Agency), its focus on dissemination and knowledge translation, and its timely orientation to pressing topics during and after the COVID-19 pandemic (e.g., global health diplomacy, emergency preparedness). Fellows and faculty emphasized the importance of these specific aspects of the program, and how it reaffirmed their commitment to public health and their learning from their involvement in CARE. Importantly, these aspects of the program facilitated the ability of many fellows to establish communication with other scientists within and outside of CARE, and engage other stakeholders (e.g., government officials, community partners).

The current study should be interpreted within the context of certain limitations. First, the sample size was small, but as noted above it represents a relatively large training program relative to others [28,29,30]; nonetheless, findings are not generalizable to other training programs or students not in such programs, and analyses were not conducted to look at subgroup differences due to the small sample size. Another limitation is that, like all self-report survey-based studies (including national and international surveillance systems), this study may be influenced by bias related to self-reporting. Additionally, qualitative data were collected using open-ended survey questions rather than in-depth interviews (in order to reduce the potential to influence participants to provide socially desirable answers, i.e., those indicating favorable impressions of the program); however, this approach precluded probing to gain further insights. Nonetheless, this mixed-methods approach allowed the integration of quantitative and qualitative data, providing greater depth to our findings. Also, although 100% participation was achieved among fellows, participation among faculty was lower (67%). Furthermore, analyses did not characterize faculty who participated in the evaluation survey versus those who did not, as the small sample size may have undermined confidentiality. Finally, at this point in time, evaluation could not assess all outcomes, particularly long-term outcomes; thus, future evaluations are needed to assess these outcomes over time, particularly using additional objective measures (e.g., publications, grants awarded, career trajectories).

## 5. Conclusions

Fellows and faculty perceived great benefits of CARE participation, and early objective indicators highlight positive trends in certain short- and intermediate-term outcomes, such as trajectories in knowledge/skills acquisition, research project completion, dissemination of findings, and degrees earned. Fellows and faculty provided insights regarding the importance of such research training programs for their country and for advancing global health efforts more broadly. Findings underscore the importance of research capacity building programs for LMICs, particularly to nurture particular skills (e.g., knowledge dissemination and translation, global health diplomacy, emergency preparedness, methods/analysis). This study also highlights the need to engage researchers at all career stages and across disciplines and professional backgrounds to build a robust network of researchers in small countries like Georgia, as well as the potentially key roles alumni may have in enhancing global health training opportunities and addressing pressing public health problems and disparities.

## Figures and Tables

**Table 1:** CARE activities.

Year	2019		2020				2021				2022				2023				2024				2025			
Quarter	3	4	1	2	3	4	1	2	3	4	1	2	3	4	1	2	3	4	1	2	3	4	1	2	3	4
Entry of each fellow cohort																										
MPH cohorts (cohort number)					1				2																	
PhD cohorts (cohort number)							1				2				3				4							
CARE meetings ^a^					x			x		x		x	x		x		x			x	x			x		
Trainings/courses																										
Integrated additional NCD content into existing courses					x	x				x	x															
Environmental health (EH)								EH				EH				EH				EH						
Research methods (RM)								RM				RM				RM				RM						
Advanced data analysis (DA)												DA				DA				DA						
Global health diplomacy (GH)																					GH					
Emergency preparedness (EP)																							EP			
Grant writing (GW)																								GW		
Ongoing activities																										
Mentored research					x	x	x	x	x	x	x	x	x	x	x	x	x	x	x	x	x	x	x	x	x	x
Fellow Club meetings						x	x	x	x	x	x	x	x	x	x	x	x	x	x	x	x	x	x	x	x	x
Responsible conduct of research					x	x	x	x	x	x	x	x	x	x	x	x	x	x	x	x	x	x	x	x	x	x
Mentorship in scientific/grant writing								x	x	x	x	x	x	x	x	x	x	x	x	x	x	x	x	x	x	x

Notes: CARE: Clean Air Research and Education program. EH: environmental health. NCDs: noncommunicable diseases. ^a^ Meetings were held in October 2020 (via Zoom among US-based faculty/leadership, due to COVID-19); June 2021; November 2021; June 2022; September 2022; March 2023; October 2023; May 2024; October 2024; and May 2025.

**Table 2:** Descriptive analyses characterizing fellows and faculty involved in CARE.

Variable	Fellows		Faculty	
	*n* = 23		*n* = 10	
	*n*	%	*n*	%
Age (M, SD)	37.63	8.18	47.67	11.91
Sex				
Male	5	21.7	4	40.0
Female	18	78.3	6	60.0
Institution (for training or as faculty)				
Tbilisi State Medical University	9	39.1	2	20.0
University of Georgia (Tbilisi)	14	60.9	4	40.0
US-based	--	--	4	40.0
Program				
MPH	4	17.4	--	--
PhD	19	82.6	--	--
Year began in the program				
2019	0	0.0	2	20.0
2020	2	8.7	1	10.0
2021	5	21.7	2	20.0
2022	7	30.4	3	30.0
2023	7	30.4	2	20.0
2024	2	8.7	0	0

Notes: CARE: Clean Air Research and Education program. M: mean. SD: standard deviation.

**Table 3:** Thesis/dissertation topics and populations of interest among CARE fellows (*n* = 23).

Variable	*n*	%
Thesis/dissertation topics (check all that apply)		
Tobacco use and/or exposure	10	43.5
Air pollution	5	21.7
Respiratory outcomes	5	21.7
Lead exposure	3	13.0
Cancer-related outcomes (e.g., diagnosis, treatment, survival)	3	13.0
Cardiovascular outcomes	3	13.0
Water, sanitation, and hygiene	2	8.7
Nutrition	2	8.7
Reproductive outcomes	2	8.7
Mental or cognitive health outcomes	2	8.7
Toxicology	2	8.7
Other ^a^	2	8.7
Populations involved in thesis/dissertation (check all that apply)		
General adult population	11	47.8
Women	6	26.1
Men	3	13.0
Children	5	21.7
Clinical/patient populations	3	13.0
Healthcare providers	3	13.0
Specific geographic populations	3	13.0
Other ^b^	4	17.4

Notes: CARE: Clean Air Research and Education program. ^a^ Other environmental pollutants, all-cause mortality, etc. ^b^ College students, etc.

**Table 4:** Thesis/dissertation study designs and sources of data among CARE fellows (*n* = 23).

Variable	*n*	%
Thesis/dissertation data (check all that apply)		
Primary data collection/analysis	11	47.8
Secondary data analysis	6	26.1
Both	6	26.1
Thesis/dissertation primary data sources (check all that apply)		
Survey data	10	43.5
Focus group data	3	13.0
Qualitative interview data	1	4.3
Biological assessments	3	13.0
Thesis/dissertation study design (check all that apply)		
Cross-sectional study	16	69.6
Longitudinal study	6	26.1
Case control study	4	17.4
Communication about thesis/dissertation research (check all that apply)		
Other scientists affiliated with CARE	11	47.8
Other scientists not affiliated with CARE	8	34.8
Government officials (e.g., Ministry of Health)	7	30.4
Community partners (e.g., local community health agencies)	8	34.8
Local non-governmental organizations or other advocacy organizations	1	4.3

Notes: CARE: Clean Air Research and Education program.

**Table 5:** CARE resources used and their perceived helpfulness among fellows (*n* = 23).

Variable	*n*	%
CARE resources used (check all that apply)		
Funds to support conducting thesis/dissertation research	18	78.3
Funds to support attending scientific conferences	8	34.8
Funds to support specific trainings outside of your university	5	21.7
Funds to support publication fees	6	26.1
Helpfulness/importance of CARE resources ^a^	**M**	**SD**
Mentorship from Georgia- and US-based mentors	3.96	0.20
Semi-annual CARE meetings	3.75	0.44
Fellow Club meetings	3.45	0.80
Special training in environmental health	3.83	0.39
Additional training in research/analytic methods	3.87	0.34
Funds to support conducting thesis/dissertation research	4.00	0.00
Funds to support attending scientific conferences	4.00	0.00
Funds to support specific trainings outside of your university	4.00	0.00
Funds to support publication fees	3.89	0.33

Notes: CARE: Clean Air Research and Education program. M: mean. SD: standard deviation. ^a^ 1 = not at all, 2 = a little, 3 = somewhat, 4 = a lot.

**Table 6:** CARE resources perceived most crucial to sustain and perceived impact.

Variable	Fellows		Faculty	
	*n* = 23		*n* = 10	
	*n*	%	*n*	%
CARE components most crucial to sustain (check all that apply)				
Structured mentor/mentee relationships	22	95.7	8	80.0
Involvement of US-based mentors	22	95.7	7	70.0
Professional development (e.g., semi-annual research meetings)	15	65.2	6	60.0
Instruction/support for:				
Finding research funding, etc.	21	91.3	7	70.0
Identifying dissemination channels (i.e., conferences, journals)	16	69.6	6	60.0
Preparing publications, abstracts, presentations, etc.	21	91.3	7	70.0
Enhanced training or key topics in:				
Environmental health	13	56.5	7	70.0
Noncommunicable disease prevention	12	52.2	7	70.0
Global health diplomacy	13	56.5	7	70.0
Research methods/data analysis	15	65.2	9	90.0
Responsible conduct of research	12	52.2	7	70.0
CARE impact ^a^	**M**	**SD**		
CARE has:				
Enhanced my research skills	4.75	0.44	--	--
Exposed me to learning I would not have had otherwise	4.63	0.65	--	--
Exposed me to learning important for my career	4.71	0.55	--	--
Facilitated professional relationships for future collaborations	4.33	0.76	--	--
Helped forge professional relationships that aided my research	4.42	0.65	--	--
Enhanced my interest in EH and/or NCDs	4.63	0.65	--	--
Enhanced my commitment to public health research	4.58	0.72	--	--
The mentorship has met my needs	4.58	0.65	--	--
I was as productive as possible in accomplishing my work	4.08	1.02	--	--
My mentors were invested in my success	4.63	0.65	--	--
Before CARE, I did not think of myself as an EH/NCD researcher	3.71	1.46	--	--
Now, I think of myself as an EH and/or NCD researcher	4.22	0.85	--	--
Five years from now, I will be conducting research in EH and NCDs	4.47	0.78	--	--

Notes: CARE: Clean Air Research and Education program. EH: environmental health. NCDs: noncommunicable diseases. M: mean. SD: standard deviation. ^a^ 1 = strongly disagree; 2 = disagree; 3 = neutral; 4 = agree; 5 = strongly agree.

## Data Availability

Data will be made available upon request to the corresponding author.

## Supplementary Materials

The following supporting information can be downloaded at https://www.mdpi.com/article/10.3390/ijerph22091433/s1. Supplementary File S1: Evaluation survey; Supplementary Table S1: Reactions to specific CARE trainings and activities among fellows; Supplementary Table S2: Fellow and faculty responses to open-ended evaluation questions; Supplementary Table S3: Summary of available data addressing short-, intermediate-, and long-term outcomes and achievements to date; Supplementary Figure S1: Conceptual framework for CARE program training in environmental health (EH) and noncommunicable disease (NCD) research; Supplementary Figure S2: Evaluation framework for CARE program, including inputs, activities and support, and outcomes.

## Abbreviations

The following abbreviations are used in this manuscript:

CARE	Clean Air Research and Education
EH	Environmental health
FIC	Fogarty International Center
MPH	Master’s of Public Health
LMIC	Low- and middle-income countries
NCD	Noncommunicable disease
NIH	National Institutes of Health
PhD	Doctorate of Philosophy
