# Supplementary material for: Evaluating a Research Training Program in Environmental Health and Noncommunicable Diseases in Georgia
Source: Int J Environ Res Public Health. 2025 Sep 14;22(9):1433. doi: 10.3390/ijerph22091433 (PMC12469674; doi:10.3390/ijerph22091433)
Supplement: Supplementary file 1 [file ijerph-22-01433-s001.zip › ijerph-3816660-supplementary.pdf]

Supplementary File S1: Evaluation of CARE Program in Georgia, 2025

We would like to ask for your feedback regarding the Fogarty D43 CARE program. This information is crucial for us to demonstrate the importance of Fogarty funding for initiatives like this, so please consider this evaluation carefully.

1) Are/were you a faculty member/mentor or a fellow/trainee?*
( ) Faculty member/mentor
( ) Fellow/trainee

2) What is your age?*
____

3) At what institution are you currently employed?*
( ) Tbilisi State Medical University
( ) University of Georgia
( ) Georgia National Center for Disease Control
( ) Georgia National Environmental Agency
( ) Emory University
( ) George Washington University
( ) Not currently employed
( ) Other; please specify: _________________

4) When did you begin participating in the program (as a faculty member or fellow)?*
( ) 2019
( ) 2020
( ) 2021
( ) 2022
( ) 2023
( ) 2024
( ) 2025

Page entry logic: This page will show when: #1 Question "Are/were you a faculty member/mentor or a fellow/trainee?" is one of the following answers ("Fellow/trainee")

Fellows I

5) Are/were you in an MPH program or the PhD program?*
( ) MPH
( ) PhD

6) Are/were you a student at TSMU or UG?*
( ) TSMU
( ) UG

Some core aspects of this program have included the annual meetings, fellow club meetings, special courses, mentorship, and research support. Below we ask about each of these components.

7) These questions ask about the Environmental Health course. To what extent do you agree with the following statements:

	1=Strongly disagree	2=Disagree	3=Neutral	4=Agree	5=Strongly agree	Not applicable
I learned a great deal in this course.	( )	( )	( )	( )	( )	( )
The material challenged and stimulated my thinking.	( )	( )	( )	( )	( )	( )
The material was relevant to my career goals.	( )	( )	( )	( )	( )	( )
Overall, the instructor was knowledgeable and clearly presented explanations of important concepts.	( )	( )	( )	( )	( )	( )

8) Please feel free to add any additional comments about the Environmental Health course.

____

____

____

____

9) These questions ask about the Global Health Diplomacy course. To what extent do you agree with the following statements:

	1=Strongly disagree	2=Disagree	3=Neutral	4=Agree	5=Strongly agree	Not applicable
I learned a great deal in this course.	( )	( )	( )	( )	( )	( )
The material challenged and stimulated my thinking.	( )	( )	( )	( )	( )	( )
The material was relevant to my career goals.	( )	( )	( )	( )	( )	( )
Overall, the instructor was knowledgeable and clearly presented explanations of important concepts.	( )	( )	( )	( )	( )	( )

10) Please feel free to add any additional comments about the Global Health Diplomacy course.

____
____
____
____

11) These questions ask about the Sociological Research Methods course. To what extent do you agree with the following statements:

	1=Strongly disagree	2=Disagree	3=Neutral	4=Agree	5=Strongly agree	Not applicable

I learned a great deal in this course.	( )	( )	( )	( )	( )	( )
The material challenged and stimulated my thinking.	( )	( )	( )	( )	( )	( )
The material was relevant to my career goals.	( )	( )	( )	( )	( )	( )
Overall, the instructor was knowledgeable and clearly presented explanations of important concepts.	( )	( )	( )	( )	( )	( )

12) Please feel free to add any additional comments about the Sociological Research Methods course.

____
____
____
____

13) These questions ask about the Data Analysis course. To what extent do you agree with the following statements:

	1=Strongly disagree	2=Disagree	3=Neutral	4=Agree	5=Strongly agree	Not applicable
I learned a great deal in this course.	( )	( )	( )	( )	( )	( )
The material challenged and	( )	( )	( )	( )	( )	( )

stimulated my thinking.						
The material was relevant to my career goals.	( )	( )	( )	( )	( )	( )
Overall, the instructor was knowledgeable and clearly presented explanations of important concepts.	( )	( )	( )	( )	( )	( )

14) Please feel free to add any additional comments about the Data Analysis course.

____
____
____
____

15) These questions ask about the Responsible Conduct of Research seminars. To what extent do you agree with the following statements:

	1=Strongly disagree	2=Disagree	3=Neutral	4=Agree	5=Strongly agree	Not applicable
I learned a great deal in this course.	( )	( )	( )	( )	( )	( )
The material challenged and stimulated my thinking.	( )	( )	( )	( )	( )	( )
The material was relevant to my career goals.	( )	( )	( )	( )	( )	( )

Overall, the instructor was knowledgeable and clearly presented explanations of important concepts.	( )	( )	( )	( )	( )	( )

16) Please feel free to add any additional comments about the Responsible Conduct of Research seminars.

____
____
____
____

17) These questions ask about the annual/semi-annual in-person Fogarty meetings. To what extent do you agree with the following statements:

	**1=Strongly disagree**	**2=Disagree**	**3=Neutral**	**4=Agree**	**5=Strongly agree**	**Not applicable**
I learned a great deal at the annual Fogarty meetings.	( )	( )	( )	( )	( )	( )
The meetings challenged and stimulated my thinking.	( )	( )	( )	( )	( )	( )
The meetings were relevant to my career goals.	( )	( )	( )	( )	( )	( )

The meetings helped me advance my research.	( )	( )	( )	( )	( )	( )
The meetings helped me develop my connections with the faculty and other fellows.	( )	( )	( )	( )	( )	( )

18) Please share your final thoughts on the annual/semi-annual Fogarty meetings.

____
____
____
____

19) Copy of These questions ask about the Fellow Club meetings with Lela and Amiran. To what extent do you agree with the following statements:

	**1=Strongly disagree**	**2=Disagree**	**3=Neutral**	**4=Agree**	**5=Strongly agree**	**Not applicable**
The Fellow Club meetings challenged and stimulated my thinking.	( )	( )	( )	( )	( )	( )
The meetings helped me advance	( )	( )	( )	( )	( )	( )

my research.						
The meetings helped me develop my connections with the faculty and other fellows.	( )	( )	( )	( )	( )	( )

20) Please share your final thoughts on the Fellow Club meetings.

____
____
____
____

Page entry logic: This page will show when: #1 Question "Are/were you a faculty member/mentor or a fellow/trainee?" is one of the following answers ("Fellow/trainee")

Fellows II

Below we ask about the content and methods used in your dissertation research. We understand that you may engage in several separate "studies" as part of your dissertation research, so ensure that you "check all that apply" to any of your dissertation-related studies.

Logic: Show/hide trigger exists.

21) Does your research involve primary data collection/analysis (i.e., collecting your own data, for example, your own survey data), secondary data analysis (i.e., analyzing existing data, for example, national surveillance data or medical chart review), or both? (Check all that apply.)*

[ ] Primary data collection/analysis
[ ] Secondary data analysis
[ ] Both
[ ] Other; please specify: __________________

Logic: Hidden unless: #21 Question "Does your research involve primary data collection/analysis (i.e., collecting your own data, for example, your own survey data), secondary data analysis (i.e., analyzing existing data, for example, national surveillance data or medical chart review), or both? (Check all that apply.)" is one of the following answers ("Primary data collection/analysis")

22) What types of primary data collection does your study involve? (Check all that apply.)*

[ ] Survey data; please specify population(s): __________________

[ ] Focus group data; please specify population(s): _________________
[ ] Qualitative interview data; please specify population(s): _________________
[ ] Biological assessments; please specify type(s) and population(s): _________________
[ ] Other; please specify: _________________

Logic: Hidden unless: #21 Question "Does your research involve primary data collection/analysis (i.e., collecting your own data, for example, your own survey data), secondary data analysis (i.e., analyzing existing data, for example, national surveillance data or medical chart review), or both? (Check all that apply.)" is one of the following answers ("Secondary data analysis")

23) What types of secondary data does your study involve? (Check all that apply.)*
[ ] NEA data; please specify nature of the data: _________________
[ ] Cancer registry data; please specify nature of the data: _________________
[ ] State lead exposure data; please specify nature of the data: _________________
[ ] Birth registry data; please specify nature of the data: _________________
[ ] Other; please specify: _________________

24) Please indicate the study designs you use in your dissertation research, in any part or study involved. (Check all that apply.)*
[ ] Cross-sectional study
[ ] Longitudinal study
[ ] Case control study
[ ] Randomized controlled trial
[ ] Other; please specify: _________________

25) Please indicate the topics covered in your dissertation. Provide some information about your measures and note how they are considered in your analyses, for example, as independent variables (i.e., predictors) or dependent variables (i.e., outcomes). (Check all that apply.)*
[ ] Nutrition
[ ] Tobacco use and/or exposure (including secondhand smoke)
[ ] Air pollution
[ ] Lead exposure
[ ] Toxicology
[ ] Water, sanitation, and hygiene
[ ] Cancer-related outcomes (e.g., diagnosis, treatment, treatment outcomes, survival)
[ ] Reproductive outcomes
[ ] Cardiovascular outcomes
[ ] Respiratory outcomes
[ ] Mental or cognitive health outcomes
[ ] Other; please specify: _________________

26) Please indicate the Georgia-based populations of interest in your dissertation research, in any part or study involved. (Check all that apply.)*
[ ] General adult population; please specify: _________________
[ ] Women; please specify: _________________
[ ] Men; please specify: _________________
[ ] Children; please specify: _________________
[ ] Clinical/patient populations; please specify: _________________

[ ] Healthcare providers; please specify: _________________
[ ] Specific geographic populations; please specify: _________________
[ ] Other; please specify: _________________

27) During the duration of the Fogarty program, indicate whether you have had email, telephone, or in-person communication with the following people or entities to discuss your Fogarty project. (Check all that apply.)*
[ ] Other Scientists Affiliated with the Fogarty Project
[ ] Other Scientists not Affiliated with the Fogarty Project
[ ] Government Officials, including Ministers (e.g., Minister of Health)
[ ] Community Partners (e.g., local community health agencies)
[ ] Television Media
[ ] Newspaper Media
[ ] Local NGOs or other advocacy organizations
[ ] Other; please specify: _________________
[ ] None of the above

28) So far, what resources from the Fogarty program have you used? (Check all that apply.)*
[ ] Funds to support conducting your research
[ ] Funds to support attending conferences
[ ] Funds to support specific trainings outside of your university
[ ] Funds to support publication fees
[ ] Other; please specify: _________________
[ ] None of the above

Page entry logic: This page will show when: #1 Question "Are/were you a faculty member/mentor or a fellow/trainee?" is one of the following answers ("Fellow/trainee")

Fellows III

29) So far, how helpful or important to you have the following resources from the Fogarty program been?*

	**1=Not at all**	**2=A little**	**3=Somewhat**	**4=Very**	**Not applicable/Have not used**
Mentorship from Georgia- and US-based mentors	( )	( )	( )	( )	( )
Annual Fogarty meetings	( )	( )	( )	( )	( )
Fellow club meetings	( )	( )	( )	( )	( )

Special training in environmental health	( )	( )	( )	( )	( )
Additional training in research/analytic methods (e.g., at UG or via Institute of Social Studies and Analysis)	( )	( )	( )	( )	( )
Funds to support conducting your research	( )	( )	( )	( )	( )
Funds to support attending conferences	( )	( )	( )	( )	( )
Funds to support specific trainings outside of your university	( )	( )	( )	( )	( )
Funds to support publication fees	( )	( )	( )	( )	( )

30) To what extent do you agree with the following statements:*

	**1=Strongly disagree**	**2=Disagree**	**3=Neutral**	**4=Agree**	**5=Strongly agree**	**Not applicable**
The Fogarty program has enhanced my research skills.	( )	( )	( )	( )	( )	( )
The Fogarty program has exposed me to learning	( )	( )	( )	( )	( )	( )

that I would not have had otherwise.						
The Fogarty program has exposed me to learning that is important for my career.	( )	( )	( )	( )	( )	( )
The Fogarty program has helped me forge professional relationships that may lead to future collaborative research.	( )	( )	( )	( )	( )	( )
The Fogarty program has helped me forge professional relationships that has aided me in conducting my research.	( )	( )	( )	( )	( )	( )
The Fogarty program has enhanced my interest in Environmental Health and/or Non-communicable Diseases.	( )	( )	( )	( )	( )	( )

The Fogarty program has enhanced my commitment to public health research.	( )	( )	( )	( )	( )	( )
The mentorship I have received through the Fogarty program has met my needs.	( )	( )	( )	( )	( )	( )
I feel like I was as productive as possible in accomplishing the work I outlined for myself in the Fogarty program.	( )	( )	( )	( )	( )	( )
I feel like my mentors were invested in my success.	( )	( )	( )	( )	( )	( )
Before the Fogarty grant, I did not think of myself as an Environmental Health and/or Non-communicable Diseases researcher.	( )	( )	( )	( )	( )	( )

Now, I think of myself as an Environmental Health and/or Non-communicable Diseases researcher.	( )	( )	( )	( )	( )	( )
Five years from now, I will be conducting research in Environmental Health and Non-communicable Diseases.	( )	( )	( )	( )	( )	( )

31) How has your participation in the Fogarty D43 CARE program helped you achieve your goals?*

____
____
____
____

Faculty and Fellows

32) What components of the Fogarty program do you think are most crucial to sustain beyond the grant funding period? (Check all that apply.)*

[ ] Structured mentor/mentee relationships
[ ] Involvement of US-based mentors
[ ] Professional development activities, such as annual research meetings, regular meetings to report on research progress (similar to current Fellow club meetings)
[ ] Instruction/support in finding research funding for dissertations, etc.
[ ] Instruction/support in identifying appropriate dissemination channels (i.e., conferences, journals)
[ ] Instruction/support in preparing publications, abstracts, presentations, etc.
[ ] Certain key topics in environmental health
[ ] Certain key topics in non-communicable disease prevention
[ ] Certain key topics in global health diplomacy
[ ] Enhanced training in research methods and data analysis
[ ] Enhanced training in responsible conduct of research
[ ] Other; please specify: _________________

33) What have you learned about yourself as a result of being a mentor or mentee in this program?

____
____
____
____

34) What about participating in the Fogarty D43 CARE program has been most valuable to you?

____
____
____
____

35) If a funder were to ask you why this Fogarty program or other similar programs are important to your country, what would you say?

____
____
____
____

36) If a funder were to ask you why this Fogarty program or other similar programs are important to global health, what would you say?

____
____
____
____

37) What would you suggest or change about this overall Fogarty program?

____
____
____
____

38) Is there anything else that you believe the people running the program – either TSMU, UG, the US team, or Fogarty International Center – should know about your experience with the project?

____
____
____
____

Thank You!

Thank you for completing this evaluation. PLEASE follow THIS LINK and provide YOUR NAME so that we know you have completed the evaluation. (Your name will be stored separately from your evaluation data.): [link]

Supplementary Table S1. Reactions to specific CARE trainings and activities among fellows (n=23)

**Variable^**	**M**	**SD**
Environmental health	4.77	0.68
I learned a great deal in this course	4.73	0.67
The material challenged and stimulated my thinking	4.81	0.63
The material was relevant to my career goals	4.68	0.63
Overall, the instructor was knowledgeable and clearly presented explanations of important concepts	4.85	0.78
Global health diplomacy	4.64	0.89
I learned a great deal in this course	4.60	0.91
The material challenged and stimulated my thinking	4.58	0.93
The material was relevant to my career goals	4.64	0.86
Overall, the instructor was knowledgeable and clearly presented explanations of important concepts	4.72	0.84
Research methods	4.10	1.14
I learned a great deal in this course	4.00	1.17
The material challenged and stimulated my thinking	4.00	1.17
The material was relevant to my career goals	4.06	1.14
Overall, the instructor was knowledgeable and clearly presented explanations of important concepts	4.35	1.06
Data analysis	4.50	1.04
I learned a great deal in this course	4.50	1.04
The material challenged and stimulated my thinking	4.44	1.04
The material was relevant to my career goals	4.44	1.04
Overall, the instructor was knowledgeable and clearly presented explanations of important concepts	4.61	1.04
Responsible conduct of research	4.57	0.69
I learned a great deal in this course	4.48	0.75
The material challenged and stimulated my thinking	4.63	0.63
The material was relevant to my career goals	4.52	0.64
Overall, the instructor was knowledgeable and clearly presented explanations of important concepts	4.63	0.74
Semi-annual CARE fellow/faculty meetings	4.51	0.93
I learned a great deal at the semi-annual CARE meetings	4.41	0.84
The meetings challenged and stimulated my thinking	4.44	0.97
The meetings were relevant to my career goals	4.48	0.94
The meetings helped me advance my research	4.44	1.05
The meetings helped me develop my connections with the faculty and other fellows	4.70	0.82
Fellow Club meetings	4.42	0.93
The Fellow Club meetings challenged and stimulated my thinking	4.42	0.88
The meetings helped me advance my research	4.38	0.92
The meetings helped me develop my connections with the faculty and other fellows	4.46	0.98

Notes: ^1=strongly disagree; 2=disagree; 3=neutral; 4=agree; 5=strongly agree; 99=n/a. Emergency preparedness course not evaluated, as it occurred after the February 2025 evaluation survey.

Supplementary Table S2. Fellow and faculty responses to open-ended evaluation questions

Fellows	Faculty
**What have you learned about yourself from being a mentor or mentee in this program?**	
I have gained insight into the value of humility and openness to learning from others. These experiences have strengthened my leadership and collaboration skills, and have given me a deeper understanding of the impact of mentorship in academic and professional development.	I have learned that mentoring is an individual endeavor and is not a one size fits all approach. Spending time to better understand your mentee, their situation, challenges, concerns, etc. is critical to developing a successful mentor/mentee relationship.
I realized that with the right guidance and support, I can overcome obstacles and improve my research skills. I have become more organized and better at solving problems, which helps me work through research challenges. I also learned to be more patient and to see difficulties as learning opportunities rather than failures. Through this experience, I have gained more confidence in myself as a researcher. This has encouraged me to set bigger goals and keep improving in research and academia.	It was a unique chance to explore new approaches to public health and gain new knowledge about important environmental health issues and challenges. The process of collaborating with colleagues was the most important benefit in this project.
It has helped me recognize my ability to navigate complex research challenges with resilience and adaptability. I have learned that I thrive in collaborative environments where mentorship and peer support enhance my growth. The program has also reinforced my passion for bridging research and policy to drive meaningful public health improvements. Additionally, I have gained confidence in my leadership and project management skills, realizing my potential to contribute to global health initiatives. This experience has further motivated me to mentor others and share the knowledge I have gained.	This program was a great opportunity for both mentors and mentees. We all learn from each other many things, among them I believe most important is to see how structured and well-designed research plan works and how well-prepared mentees improve.
It has taught me the importance of resilience, adaptability, and continuous learning. I've discovered my ability to tackle complex problems, manage challenges, and work collaboratively with diverse teams. It has also helped me recognize areas for personal growth, particularly in leadership and time management, and has strengthened my commitment to pursuing impactful research.	
It has taught me a lot about myself. I've learned how important it is to listen, ask questions, and be open to feedback. I've become more confident in my abilities and better at reflecting on my strengths and areas for growth. This experience has shown me how valuable it is to have guidance and support to help me grow professionally and personally.	
I have learned to better understand my strengths, set clear goals, and effectively support others while growing personally and professionally.	
This program likely instilled a deeper sense of commitment to continuous growth and learning in me, which I already had before. I have discovered how much I enjoy diving deep into	

research or tackling complex problems, fueling my passion for global health or scientific inquiry.	
The program offered structured educational opportunities, enhancing skills in research methodology, data analysis, and scientific writing, which are critical for producing high-quality research in public health.	
**What about participating in CARE has been most valuable to you?**	
**The most important aspect for me has been:**	
The opportunity to connect with a diverse group of mentors, researchers, and professionals. This collaboration led to new partnerships, resource sharing, and insights into global health research and practices.	Networking - I met amazing people and worked with great team from US, it was really productive to work with UG and Us-based mentors together.
Exposure to cutting-edge research and the opportunity to collaborate with experts in global health and environmental studies. This has greatly enhanced my understanding of the complex relationship between environmental factors and public health. Additionally, the program's focus on data analysis and the mentorship opportunities have strengthened my research skills and provided me with the tools to make significant contributions to my field. Overall, the program has been a pivotal step in advancing my academic and professional goals.	Working with international partners. It has been a hands-on learning experience that the environmental health issues are truly global but may manifest in different ways given the international circumstances.
The opportunity to build a strong professional network with both local and international experts in public health and epidemiology. Engaging with leading researchers, policymakers, and practitioners has broadened my perspective and opened doors for future collaborations. Additionally, the program has provided a platform to exchange knowledge, gain insights from diverse healthcare systems, and apply innovative approaches to my research. These connections and learning experiences have been instrumental in enhancing my expertise and advancing my career in public health.	It was very important for me to cooperate with foreign partners, to share their opinions regarding the research process and methods.
The opportunity to conduct my own research and develop a scientific paper under the guidance of experienced mentors. Their expertise has been crucial in improving my research skills, and the program has provided me with a platform for professional growth and networking.	
The opportunity to engage in meaningful global health research, receive guidance from experienced mentors, and build a professional network with researchers from diverse backgrounds.	
Without the support of the CARE program, I wouldn’t have had opportunity to pursue a PhD.	
**If a funder were to ask you why CARE or similar programs are important to your country, what would you say?**	
Georgia, as a post-Soviet country, faces a significant challenge. The declared desire of our country's population is full integration into multilateral structures (NATO, European Union), and the	Since Georgia is really lacking capacity in well-designed research, the scientific aspect at universities need to be improved. Even though we are trying

support of education and science by such foundations will soon bring our country closer to its goal.	very hard, I am certain that transferring knowledge and sharing experience is vital in conducting research and also creating a good basis for further development.
The CARE program and similar initiatives are absolutely crucial to my country because they foster the development of skilled researchers who can address local and global health challenges. These programs help build research capacity, enhance collaboration with international experts, and promote evidence-based solutions to improve public health. By investing in these programs, my country can strengthen its healthcare systems, advance scientific knowledge, and contribute to global health efforts.	The existence of such type of projects allows PhD students to develop in accordance with international standards, to acquire new knowledge and skills, which are necessary for future scientific activities. Often PhD students have financial barriers, so the existence of the program and financial support in the process of research and publication is a key issue.
Programs like this are vital because they help develop local expertise in global health, which is essential for tackling health challenges in my country. They also create opportunities for collaboration and knowledge exchange, which strengthens our research and health systems.	Georgia, as a lower-middle-income country, greatly benefits from programs like the CARE program, which provide essential training and capacity-building opportunities for young professionals. These programs equip researchers and public health experts with the skills and knowledge needed to address critical health challenges, strengthen health systems, and contribute to evidence-based policymaking. Additionally, they foster international collaborations, enabling Georgian professionals to engage with global experts, access cutting-edge research methodologies, and implement best practices locally. Investing in such initiatives is crucial for developing a strong public health workforce that can drive sustainable improvements in healthcare and population health in Georgia.
The CARE program and similar initiatives are crucial for my country as they provide vital training and resources to address pressing health and environmental challenges. Programs like these equip researchers with the skills to conduct high-quality research, which is essential for informing policy decisions and improving public health outcomes. They also foster international collaborations that enhance the capacity of local institutions and researchers to tackle issues such as air pollution, disease prevention, and environmental health. By strengthening research networks and building local expertise, these programs play a key role in advancing sustainable development and improving the well-being of our population.	
Programs like this are crucial for strengthening Georgia's capacity to conduct high-quality research. As a small country, Georgia benefits from partnerships that provide access to international expertise, resources, and cutting-edge methodologies, enabling local researchers to address both national and global health challenges effectively.	
Programs like this invest in the development of local researchers and public health professionals, equipping them with the skills and knowledge needed to address health challenges. Moreover, it fosters international partnerships, allowing researchers in my country to collaborate with global experts. These connections open doors to new knowledge, resources, and opportunities, enabling innovative approaches to health challenges.	
This program was crucial for building capacities to create new evidence in public health and environmental health, to inform	

policy decisions for my country, and to have a positive regional and global impact.	
**If a funder were to ask you why CARE or similar programs are important to global health, what would you say?**	
It creates new resources for developing research capacities in the fields that are relevant for current challenges. for example, we cannot advance environmental agenda only in one country, this is a global issue that requires local and regional attention and capacities, and programs like this enable scientific dialogue and create platforms for collaboration.	Similar to Q7, public health is global and we cannot take an isolationist approach. Working on the international stage ensures a strong global partnership and highlights the value of diversity and the benefits of different voices in addressing issues that impact all of us.
This program and similar initiatives are essential to global health as they provide researchers from around the world with the tools, knowledge, and support needed to tackle complex health issues that transcend borders. By focusing on areas such as environmental health, infectious diseases, and health disparities, these programs promote a collaborative, interdisciplinary approach to solving global health challenges. They also help build research capacity in low- and middle-income countries, which is vital for addressing emerging health threats and improving health outcomes globally. Ultimately, these programs strengthen global health systems by fostering knowledge exchange and driving impactful, evidence-based solutions.	The existence of the Global Health Project is important in terms of collaboration between researchers in studying the spread and characteristics of diseases globally, to make common decisions on preventive topics, as well as on environmental health issues. Publications, which will be prepared with support of the project, will significantly help scientists to get new information about the different aspects of the noncommunicable diseases in different countries.
This program and similar initiatives are essential for global health because they build research capacity, strengthen health systems, and foster international collaboration. By equipping professionals from diverse backgrounds with advanced skills in public health and epidemiology, these programs contribute to the development of evidence-based policies and innovative solutions to pressing health challenges. They also create a network of experts who can share knowledge, adapt successful interventions across different contexts, and respond effectively to emerging global health threats. Investing in such programs ultimately leads to more resilient healthcare systems and improved health outcomes worldwide.	This program covers many fields of research, since environmental and noncommunicable disease are crucial part of global health, I would say the research projects developed within the program and enhanced knowledge (including publishing articles and taking part in scientific literature) are small but still step forward to better global health.
Programs like this play an important role in strengthening global health research and capacity. Investing in these programs benefits everyone. It creates a stronger global research network, encourages knowledge exchange, and supports the development of evidence-based policies that can improve public health worldwide.	
By supporting local research and empowering scientists in low- and middle-income countries, these programs help improve health outcomes worldwide. They also promote the sharing of knowledge and best practices across borders, strengthening the global response to health challenges.	

This program is essential to global health because it promotes collaboration between researchers worldwide, facilitates the exchange of knowledge and expertise, and strengthens health systems in low- and middle-income countries.	
**What would you suggest or change about the overall CARE program?**	
The program fully responds to the challenges in Georgia. I wouldn't want to change anything.	The working framework of the program is structured, as well as the form of cooperation with universities, so no need for changes.
I would suggest: 1) expanding the program's focus to include more opportunities for interdisciplinary collaboration, bringing together experts from diverse fields such as policy, economics, and environmental health, and 2) fostering long-term partnerships and providing post-program support for career development to enhance its impact on global health research and sustainability.	I would try to ensure that the PhD students have enough time to work on their dissertation while being in the program.
I greatly appreciate the opportunities provided by the CARE program, but I believe a few enhancements could make it even more impactful. Expanding mentorship opportunities by incorporating more one-on-one guidance from global experts would provide deeper, tailored support for mentees. Additionally, increasing hands-on training in grant writing and project management could help young researchers secure funding and lead independent studies more effectively. Offering more exchange or fieldwork opportunities in different healthcare settings would also enhance practical learning. Finally, creating a structured alumni network could foster long-term collaboration and knowledge exchange among past and current participants.	I would suggest longer time period for the future projects if possible. I believe preparation stage is very important for everyone involved. Students need also to be guided from very beginning, which might not be part of their PhD but the project period should be longer.
Overall, the CARE program has been incredibly valuable, but I would suggest increasing the opportunities for long-term collaboration between fellows and their international mentors. While the program provides excellent training and resources during the fellowship period, establishing ongoing partnerships beyond the program could further enhance the impact on both personal and professional development.	The biggest barrier has been the engagement of students with their mentors and projects. Students should be much more responsible for making progress and utilizing their mentors to assist them. This means closer communication with mentors and putting suggestions into practice to move their projects forward.
**Is there anything else that you believe the people running the program – including TSMU, UG, the US team, or the US National Institutes of Health – should know about your experience with the project?**	
I am deeply grateful for the support and guidance provided by all the teams involved in the CARE program, including TSMU, UG, the US team, and NIH. My experience with the project has been transformative, and I have gained invaluable knowledge and skills that have greatly enhanced my research. One key takeaway is the importance of the cross-disciplinary approach in tackling complex global health issues, and the program's ability to foster international collaborations has been an exceptional	I hope I have fully expressed my opinion I would like to thank the participants of the program and wish them success.

asset. I would also like to highlight the ongoing mentorship and personalized support that has significantly contributed to my academic and professional growth.
I would like to express my gratitude to TSMU, UG, the US team, and NIH for their dedication to this program. My experience has been incredibly valuable, providing me with essential knowledge, skills, and networking opportunities that have significantly advanced my career. One aspect I particularly appreciate is the collaborative environment, which has allowed me to engage with both local and international experts.
The support and guidance from my mentors have been incredible and crucial in keeping me motivated and helping move forward. Everyone involved in this program has been ready to offer valuable advice and encouragement whenever I needed it. It has been an invaluable experience that I believe will have a lasting impact on my future career and research.
Through the program, I have developed advanced research skills that were crucial for my growth. This includes data analysis techniques, and gaining a deeper understanding of global health issues. The exposure to cutting-edge research approaches likely enriched my ability to carry out meaningful studies. I always will be grateful to this amazing opportunity, to be the part of this project. Thank you!

Supplementary Table S3. Summary of available data addressing short-, intermediate-, and long-term outcomes and achievements to date (*indicates ongoing/future evaluation needed to assess outcomes)

Outcomes	Achievements to Date
Short-term outcomes:	
1) Increase fellow knowledge/skills in EH, NCDs, methods/analysis, responsible conduct of research, dissemination and translation	All fellows have completed core trainings and demonstrate knowledge/skills acquisition (via coursework, research, dissemination, etc.)
2) Increase number of:<br>a) mentored trainees, mentors, and mentor research projects<br>b) degrees earned	a) CARE has enrolled 4 MPH students and 15 PhD students (100% retention) and involves 15 Georgia- and US-based mentors. Fellows all lead thesis/dissertation research, supported by Georgia/US mentor pairs.<br>*b) 4 MPH fellows due to graduate have graduated; 3 PhD fellows due to graduate in 2025 are on target to do so; subsequent cohorts also on target.
Intermediate outcomes:	
1) Increase research in EH/NCD and public health in general in Georgia, indicated by number of:<br>a) peer-reviewed publications<br>b) abstracts presented<br>c) research grant applications submitted/awarded and research projects completed	*a) Fellows are actively publishing research findings. Specific to thesis/dissertation papers alone:<br>--2 of 4 MPH theses have been published to date.<br>--Of the 15 PhD students, 9 have papers published/accepted from their dissertations (yielding 18 papers) to date.<br>*b) Fellows are actively presenting their research via abstracts at scientific/professional meetings (~24 to date).<br>*c) Given fellows' stages in their doctoral programs at this time, no fellow has submitted grant applications.
2) Enhance career development/promotion	*Fellows are serving in high-level positions and earning promotions in organizations such as the National Centers for Disease Control and Public Health, the National Environmental Agency, and other organizations under the Ministry of Health. They are assuming professor/instructor positions in public health at Tbilisi-based universities.
Long-term outcomes:	
1) Enhance infrastructure and capacity for high-quality research on EH and NCDs	*CARE has contributed to the enhancement of public health curriculum in two premier public health universities in Georgia, by enhancing existing courses to include additional NCD content and contributing additional courses in EH, global health diplomacy, emergency preparedness, methods, and analysis.<br>*CARE has also enhanced mentorship skills of current mentors and fellows who will develop into mentors for future researchers.
2) Develop a critical mass of EH/NCD researchers and increase multidisciplinary collaboration among Georgian researchers and key institutions	*Indicated by achievements above and via networking activities (e.g., CARE meetings, scientific/professional meetings), representation of fellows and faculty across various institutions, backgrounds, disciplines, and professional experiences – in public health (e.g., EH, global health, epidemiology, health behavior sciences, biostatistics), law, public administration, etc.

Notes: *Indicates evaluation is incomplete; additional data collection required to assess that outcome.

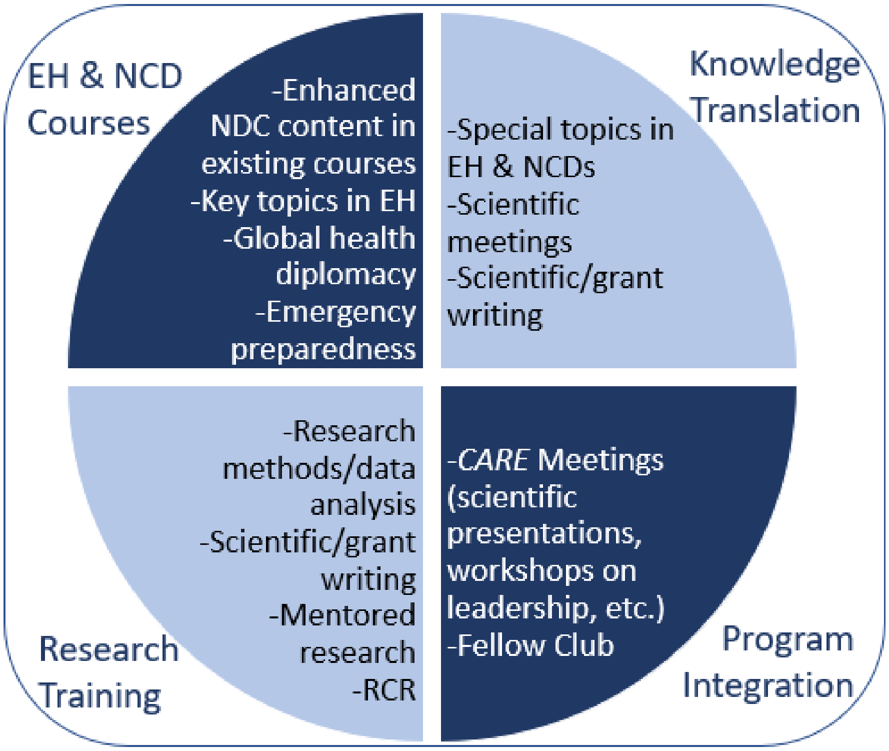

Supplementary Figure S1. Conceptual framework for CARE training program in environmental health (EH) and noncommunicable disease (NCD) research

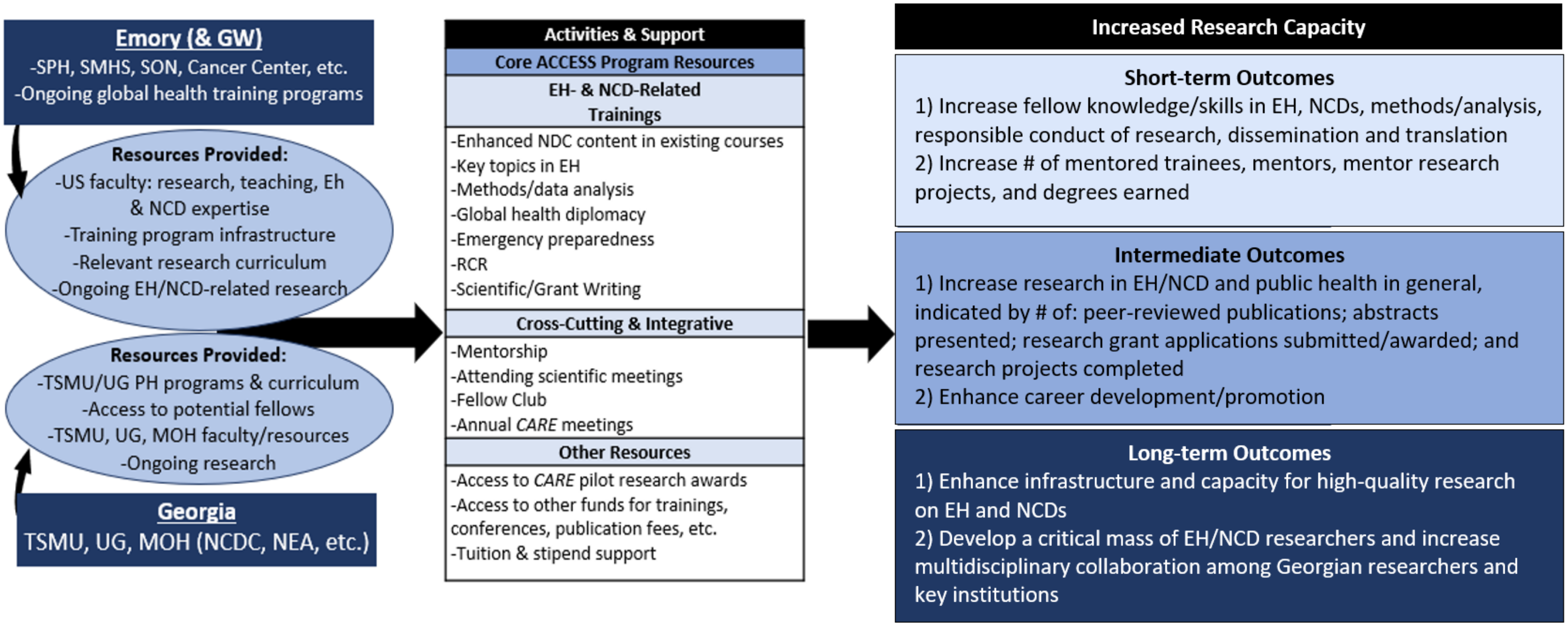

Supplementary Figure S2. Evaluation framework for CARE program, including inputs, activities and support, and outcomes
